# Standards for MicroED

**DOI:** 10.1063/4.0001185

**Published:** 2025-10-27

**Authors:** Johan Unge, Brent L Nannenga, Allen G Oliver, Tamir Gonen

**Affiliations:** 1 Umeå University, Umea, Umea, Sweden; 2 University of Notre Dame, Notre Dame, IN, USA; 3 University of California,, Los Angles, CA, USA

## Abstract

As the electron diffraction technique MicroED gains momentum and is increasingly embraced by researchers in both academia and industry, we have the opportunity to familiarize ourselves with the characteristics of MicroED data and results. The number of refined structures and their associated data is steadily growing and becoming more accessible to the scientific community, offering valuable insights into the significance and quality of MicroED-derived structures. Additionally, the growing body of experience is helping to identify best practices for the technique. Generally, MicroED maps tend to have higher resolution than what was possible with xray diffraction given the small size of the crystals. As a main observation, it is illustrative to note that for structures in Protein Data Bank (PDB) (Berman et al.), the parameters Rmerge and CC1/2 are the only parameters in this analysis where clearer differences between MicroED and X-ray data can be discerned. These parameters are related to the internal consistency of the measured intensities and describe the data before merging of the intensities and multiple data sets. For structures deposited to Cambridge Structural Database (CSD) (Groom et al.), also R1 displays larger differences, but produces reliable models. At this stage the biggest hurdle for an even wider dissemination of the MicroED technology to the scientific community is the lack of experience, infrastructure and national facilities with robust dedicated instruments for the method

